# Chemoresistance in Ovarian Cancer and Association with Circadian Rhythm

**DOI:** 10.1007/s43032-026-02093-9

**Published:** 2026-05-04

**Authors:** Fatma Seher Pektopal, Selen Kum Özşengezer, Zekiye Sultan Altun

**Affiliations:** Department of Basic Oncology, Oncology Institute, Dokuz Eylül University, Izmir, Turkey

**Keywords:** Ovarian cancer, Circadian rhythm, Chemoresistance, Cisplatin

## Abstract

Ovarian cancer (OC) accounts for a significant percentage of cancer-related deaths in women worldwide. A small proportion of ovarian cancers, usually in women under 40, originate from germ cells and stromal cells within the ovary, but the vast majority are epithelial malignancies. The preferred treatment for ovarian malignancies consists of platinum-based chemotherapy and surgical intervention.

Chemoresistance in ovarian cancer is a complex problem that significantly impedes tratment efficacy. Many pathways contribute to this resistance, including genetic factors, tumor microenvironment interactions, and lifestyle variables such as obesity. Understanding these mechanisms is crucial for developing effective treatment solutions. The circadian rhythm, or circadian clock, regulates the sleep–wake cycle, body temperature, hormone secretion, metabolism, and many physiological processes. Circadian genes are highly expressed in the ovaries, which regulate ovulation. Disruption of circadian rhythm asociated with many risk factors for ovarian cancer. This article examines the relationship between circadian rhythm and chemotherapy resistance in ovarian cancer, and reviews circadian rhythm to reduce chemotherapy resistance in this context.

## Ovarian Cancer

Ovarian cancer (OC) is the seventh most prevalent cancer type in women globally and the eighth leading cause of cancer-related deaths. [1, 2]. Although it has higher incidence rates in developed locations, it is noted to have lower rates in less developed countries. In high-income countries, the incidence has shown a declining tendency in recent years, while it has been rising in low-income countries. Consistent with the global trend, most ovarian cancer cases are diagnosed at advanced stages. This complicates tretament procedures and reduces chances for survival. In conclusion, early diagnosis, raising awareness, obesity management, and the promotion of healthy practices such as smoking cessation are crucial in combating this kind of cancer in Turkey. [1, 2].

Ovarian cancer is a malignancy with various types. A small portion of ovarian malignancies, usually in women under 40 years old, originate from germ cells and stromal cells within the ovary. [3]. A significantly larger proportion consists of epithelial malignancies. This category exhibits genetic and etiological diversity. They are classified into five main histotypes: high-grade serous, low-grade serous, endometrioid, clear cell, and mucinous tumors, each respondes differently to treatment. In addition to these invasive cancers, there are also borderline serous and mucinous neoplasms, that Show high proliferation and atypia compared to benign tumors, but are less distinct than low-grade carcinomas. The predominant histotype originates from serous tubal intraepithelial carcinoma (STIC) lesions found at the fimbrial of the fallopian tubes, whereas endometrioid and clear cell malignancies likely develop in ares of endometriosis [3, 4].

Familial history is the most important risk factor for ovarian cancer. Hereditary ovarian cancer manifests in three clinical patterns. These include regional ovarian cancer, breast-ovarian cancer syndrome, and hereditary nonpolyposis colorectal cancer (HNPCC; Lynch II) syndrome. The first two categories are linked to germline mutations in the tumor suppressor genes BRCA1 and BRCA2 (Breast Cancer Gene 1/2), while HNPCC (Hereditary Nonpolyposis Colorectal Cancer) is connected to germline mutations in the DNA mismatch repair (MMR) genes, hMLH1 (mutL homolog 1) and hMSH2 (MutS homolog 2) genes [5]. At least 10% of all epithelial ovarian tumors are genetically based. BRCA gene mutations account form more than 90% of cases, while most of the remaining 10% are associated with HNPCC. The cumulative lifetime risk of ovarian cancer is 40 to 50 percent for BRCA1 mutation carriers and 20 to 30 percent for BRCA2 mutation carriers. Germline BRCA mutation- associated ovarian tumors are typically detected at a younger ages and are characterized as high-grade advanced serous carcinomas [5].

The current advancement and use of next-generation sequencing technologies enable the simultaneous analysis of numerous cancer susceptibility genes, thereby minimizing delays and costs while improving the molecular diagnosis of hereditary tumors [6]. Identifying mutations in ovarian cancer susceptibility genes in healthy women may lead to more personalized cancer risk management through individualized clinical and radiological monitoring, chemopreventive strategies, and/or prophylactic surgery. Conversely, the discovery of mutations in individuals with ovarian cancer may reveal potential targets for biological therapies and inform treatment decisions.

The preferred treatment for ovarian cancer is platinum-based chemotherapy and surgical intervention. However, platinum resistance in ovarian cancer remains a significant medical problem. Consequently, to optimize medical benefits and improve patient outcomes, it is crucial to identify robust biomarkers and therapeutic targets in conjunction with personalized treatment [7].

## Mechanisms Involved in Chemotherapy Resistance in Ovarian Cancer

Chemothearpy resistance in ovarian cancer is a complex problem that signifcantly impedes treatment efficacy. Many pathways contribute to this resistance, including genetic factors, interactions within the tumor microenvironment, and lifestyle variables such as obesity. Understanding these mechanisms is crucial for developing effective treatment solutions.

**Fig. 1:**
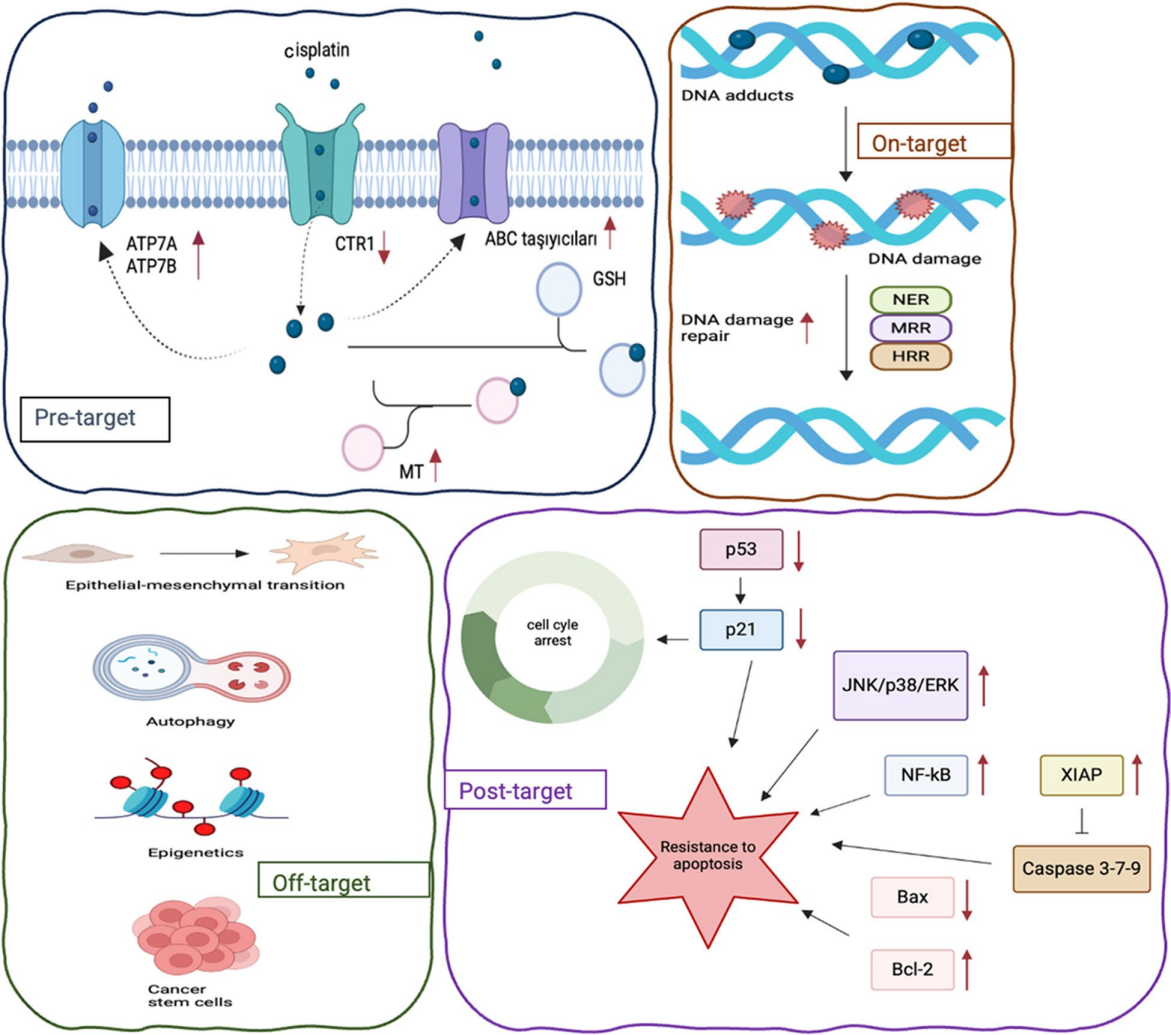
Mechanisms facilitating cisplatin resistance in ovarian cancer cells. Copper influx transporter copper transport protein 1; ABC transporters, transporters, ATP-binding cassette transporters; *ATP7A/B*, ATPase copper-transporting alpha/beta; *GSH*, glutathione; *MT*, metallothionein; *NER*, nucleotide excision repair; *MMR*, mismatch repair; *HRR*, homologous recombination repair

Cisplatin is a chemotherapeutic drug commonly employed in the management of ovarian cancer. Cisplatin (cis-[PtCl2(NH3)2], DDP) was the inaugural platinum-based complex sanctioned by the FDA (Food and Drug Administration for the treatment of several cancers, including cervical, colorectal, lung, recurrent lymphoma, and ovarian cancer. CDDP (Cisplatin (cis-[PtCl₂(NH₃)₂], DDP) primarily induces cell death by triggering a DNA damage response. Nonetheless, the effectiveness of CDDP in OC (ovarian cancer) cells diminished owing to the development of drug resistance. The mechanisms of drug resistance in ovarian cancer cells encompass reduced intracellular drug concentration, enhanced detoxification, augmented DNA repair and damage response, deregulation of apoptosis, alterations in the tumor microenvironment, and evasion of the host immune response [8]. CDDP resistance is contingent upon the variability of DNA damage and apoptotic mechanisms resulting from the non-specificity of its DNA binding. Resistance to CDDP is linked to many routes. All factors influencing the binding of CDDP to DNA and apoptosis, including pre-target resistance due to alterations occurring prior to CDDP binding to cellular targets, and on-target resistance resulting from modifications in DNA-CDDP adducts. Cisplatin resistance in ovarian cancer may arise from mutations and alterations in downstream signaling pathways that trigger apoptosis, leading to post-target resistance, as well as modifications in cellular pathways not directly associated with CDDP-induced signaling, resulting in off-target resistance [8] (Fig. 1).

### Drug Efflux Proteins

Drug efflux proteins play a crucial role in the development of treatment resistance in ovarian cancer [9]. Chemotherapeutic drugs must be delivered to the cell cytoplasm to achieve optimal therapeutic efficacy. Efflux proteins can prevent drugs from reaching the cytoplasm by expelling various drugs from the cell. The main drug efflux and membrane transporter proteins associated with resistance belong to the adenosine triphosphate-binding cassette (ABC-ATP-binding cassette) superfamily. ABC transporters use adenosine triphosphate (ATP) to transport substrates across the cell membrane. They consist of two cytoplasmic nucleotide-binding domains responsible for recognizing the transport molecules and two transmembrane domains responsible for ATP binding and hydrolysis, respectively. This large protein family is divided into seven subfamilies labeled A through G based on sequence homology. Specifically, the ABCB1 (P-glycoprotein/PgP, multidrug resistance protein 1/MDR1), ABCC1 (multidrug resistance-associated protein 1/MRP1), and ABCG2 (breast cancer resistance protein/BCRP) proteins are associated with chemotherapy resistance in ovarian cancer [9].

### Multi-drug Resistance-MDR

In various cancer forms, resistance to chemotherapy is mostly attributed to multidrug resistance in cancer cells. The MDR1 (Multidrug Resistance Protein 1) gene encodes the P-glycoprotein drug transporter. High MDR1 gene expression or P-glycoprotein levels are considered correlates of drug resistance. Resistance gene MDR1, LRP (Lung Resistance Protein), and GSTP1 (Glutathione S-transferase P1) expression levels are significantly higher in epithelial ovarian cancer patients who have not previously received chemotherapy or radiation therapy compared to patients with borderline adenoma and benign adenoma[10].

The importance of multidrug resistance-associated proteins (MRPs) in ovarian cancer is significant. Studies have shown that miRNAs (microRNAs) modulate the expression of MDR efflux transporters in the ABC superfamily in various tumors by upregulating MRP3, MRP4, and MRP5 and expressing MRP7 [11, 12].

### Tumour Microenvironment

The tumor microenvironment may contribute to recurrence and resistance to chemotherapy. Tumor-associated macrophages (TAMs) represent the predominant immune cell type within the ovarian tumor microenvironment (TME) [13]. These cells play a significant role in the progression of ovarian cancer and resistance to chemotherapy. The primary tumor-supporting role of M2-like TAMs (tumor-associated macrophages) is to activate survival signaling pathways through the release of various cytokines, chemokines, enzymes, and exosomes that facilitate microRNA access and directly increase the invasion potential and chemotherapy resistance of ovarian cancer cells [13]. It has been suggested that the complex interaction between ovarian cancer cells and tumor-associated macrophages in the tumor microenvironment is a factor contributing to treatment failure. While the role of TAMs in chemotherapy resistance is widely accepted in breast cancer, it has also been shown to be significant in ovarian cancer [14]. The main mechanisms of chemotherapy resistance in ovarian cancer associated with TAM include the tumor-supporting polarization of macrophages, their effects on survival signaling pathways, and the activation of multidrug resistance genes in cancer cells mediated by macrophages [13].

### Epigenetic Factors

Epigenetic changes, particularly DNA methylation, histone modifications, and non-coding RNA regulation, play a significant role in ovarian cancer treatment resistance. Epigenetic changes facilitate chemotherapy resistance through multiple pathways, including modulation of MRP (Multidrug Resistance-Associated Proteins) expression, alteration of the tumor microenvironment, and dysregulation of the immune response [15].

The epigenetic control of ABCB1 (P-glycoprotein/PgP, multidrug resistance protein 1/MDR1) is associated with drug transport in ovarian cancer cells. Sun et al. demonstrated that paclitaxel treatment enhanced histone H3 acetylation and specifically targeted the androgen receptor (AR) and ABCB1 promoter, resulting in the expression of the ABCB1 gene and the development of a paclitaxel resistance phenotype [16]. Wang et al. demonstrated that hnRNPA2B1 (heterogeneous nuclear ribonucleoprotein A2/B1) associates with the 5'UTR of ABCC2 mRNA, thereby increasing its translation and leading to cisplatin resistance in ovarian cancer [17]. Calcagno et al. determined that increased acetylation of histone H3 in the ABCG2 promoter underlies doxorubicin resistance [18]. These findings demonstrate that epigenetic control significantly affects resistance to ovarian cancer treatment by modifying the ABC transporter family.

ATP-binding cassette (ABC) transporters are major determinants of chemotherapy resistance in ovarian cancer by actively reducing intracellular concentrations of cytotoxic agents. ABCB1 (P-glycoprotein/MDR1) is the most extensively studied member; it is frequently overexpressed in cisplatin- and paclitaxel-resistant ovarian cancer models and functions as an ATP-dependent efflux pump that reduces drug accumulation and cytotoxicity [19, 20]. ABCC1 (MRP1) and related transporters (ABCC3, ABCC4, ABCC5, ABCC10/MRP7) similarly contribute by transporting platinum derivatives out of the cell a long with glutathione and glucuronide conjugate metabolites, while also supporting redox homeostasis and survival under chemotherapy-induced stress [21]. ABCG2 (BCRP) is highly expressed in ovarian cancer stem-like cells, where it facilitates efflux of agents such as cisplatin and topotecan and is strongly associated with the maintenance and stem cell properties and tumor recurrence [19].

The expression of these transporters is regulated through multiple layers. For example, miR-130a is upregulated in cisplatin-resistant SKOV3/CIS cells; its inhibition reduces MDR1 expression and restores cisplatin sensitivity [22]. A more comprehensive study confirmed that miR-27a and miR-130a increase P-gp expression and contribute to drug resistance in ovarian cancer [23]. Additionally, alternative polyadenylation (APA) of ABCC1 transcripts has been reported in high-grade serous ovarian cancer. This produces truncated 3′UTR variants that prevent microRNA-mediated suppression, thereby maintaining high transporter levels and conferring resistance and poor survival [24]. These findings briefly demonstrate that ABCB1, ABCC1, and ABCG2 not only function as efflux pumps but also support apoptosis escape, redox stability, and stem cell properties. Their regulation by microRNAs and APA provides further insight into the post-transcriptional mechanisms of chemotherapy resistance in ovarian cancer [19, 20, 23].

Epigenetic dysregulation plays a central role in shaping the response to chemotherapy in ovarian cancer. DNA hypermethylation of tumor suppressors such as BRCA1/2, MLH1, and RASSF1A leads to impaired DNA repair, inadequate mismatch repair, or loss of apoptotic signaling, thereby promoting platinum resistance and poor prognosis [25]. Conversely, hypomethylation of oncogenes, including HOXA9, CBX8, AGR2, and GABRP, has been associated with tumor progression and metastasis. Methylation-induced silencing of regulators in the Wnt/β-catenin pathway (SFRP5, IQGAP2, TMEM88) and TGF-β pathway (FBXO32, ABCA1, SOX2, TGFBI) further facilitates epithelial-mesenchymal transition (EMT), increased proliferation, and resistance to platinum therapy (Wang et al., 2022). Methylation changes in genes associated with EMT, such as MSX1 and LAMA3, also contribute to the emergence of cancer stem cells (CSCs), which are closely linked to drug resistance. Importantly, clinical studies with DNMT inhibitors, including azacitidine, decitabine, and guadacitabine, have shown that targeting methylation can overcome platinum resistance and enhance immune responses [26]. Histone modifications similarly affect chemotherapy sensitivity. Overexpression of histone deacetylases (HDACs), particularly HDAC1 and HDAC6, has been associated with tumor progression and poor clinical outcomes. HDAC-mediated hypoacetylation silences proapoptotic genes such as BAX and p21. Pharmacologic inhibition of HDACs has been shown to restore apoptosis and re-sensitize resistant ovarian cancer cells to cisplatin and paclitaxel [27] Non-coding RNAs add another critical layer of regulation. MicroRNAs such as miR-130a upregulate MDR1/ABCB1 and promote drug efflux, miR-214 targets PTEN to activate PI3K/AKT signaling pathway and inhibit apoptosis, and the miR-200 family controls EMT and thus modulates platinum sensitivity. Additional microRNAs, including miR-9, miR-520d-3p, miR-206, and miR-93, have been linked to chemosensitivity and prognosis [28]. Collectively, these epigenetic mechanisms converge on pathways controlling DNA repair, apoptosis, EMT, and drug efflux, offering both a mechanistic understanding and therapeutic opportunities to overcome chemotherapy resistance in ovarian cancer.

#### MicroRNAs

MicroRNAs form bidirectional regulatory loops with the circadian clock and intersect with pathways governing drug efflux and apoptosis in ovarian cancer (OC). For example, miR-142-3p is transcriptionally regulated by CLOCK:BMAL1, directly targets the 3′UTR of BMAL1, and forms a negative feedback module within the nuclear oscillator [29, 30]. In OC models, several miRNAs modulate chemoresistance by controlling ABC transporters and survival signaling: miR-130a is upregulated in cisplatin-resistant cells, and its inhibition reduces MDR1/P-glycoprotein (ABCB1) and restores cisplatin sensitivity [22]; miR-27a promotes paclitaxel resistance by increasing MDR1/P-gp, at least partly via HIPK2 [31]; and miR-186 enhances sensitivity to both paclitaxel and cisplatin by directly targeting ABCB1 [32]. In parallel, tumor-suppressive miR-34a reduces proliferation and rescues platinum sensitivity in OC by repressing HDAC1 and BCL-2–linked anti-apoptotic programs [33]. Integrating these data with multiple omics evidence of MDR1 undergoing epigenetic dysregulation of circadian genes (e.g., ARNTL/BMAL1, CLOCK, PER/CRY) epigenetic dysregulation with prognostic and therapeutic significance in OC, suggests that miRNA-clock interaction may shape chemotherapy response by affecting MDR1 upregulation and apoptosis resistance [34].

Kumar et al. illustrated that miRNAs play a role in cisplatin-resistant cells, potentially targeting numerous critical pathways, including MAPK (Mitogen-Activated Protein Kinase), TGF-β (Transforming Growth Factor Beta) signaling, actin cytoskeleton, ubiquitin-mediated proteasomal pathway, Wnt signaling, mTOR (Mechanistic Target of Rapamycin) signaling, Notch signaling, apoptosis, and various other signaling pathways. Modulating one or more of these miRNAs may represent significant targets for ovarian cancer treatment [35].

In ovarian tumors, miR-9 has been demonstrated to downregulate BRCA1 and enhance sensitivity to cisplatin and PARP (Poly (ADP-ribose) Polymerase) inhibitors by inhibiting DNA damage repair, whereas miR-490-3p has been found to reduce ABCC2/MRP2 levels and improve responsiveness to cisplatin [12].

Han et al. (20) established that hepatic leukaemia factor (HLF) expression is elevated in ovarian cancer (OC) tissues and ovarian cancer stem cells (CSCs), and that HLF governs OC cell stemness, proliferation, and metastasis. HLF (hepatic leukaemia factor) is aberrantly expressed in human cancers. The study demonstrated that HLF transcriptionally activates Yes-associated protein 1 (YAP1) expression, hence modulating the Hippo signaling system. Moreover, miR-520e was demonstrated to directly target the HLF 3′-UTR in ovarian cancer cells. The HLF/YAP1 axis was demonstrated to influence the response to carboplatin [36].

A longitudinal investigation of miRNA expression patterns in HGSOC (High-Grade Serous Ovarian Cancer) patients undergoing neoadjuvant chemotherapy (NACT) indicates that the expression levels of miR let-7G-5p, miR-199a-3p, miR-199a-5p, and miR-181a-5p are independently correlated with overall survival. Furthermore, these four miRNAs are related with platinum-based resistance and prognosis. The co-expression of P-Smad2 and miR181a-5p in surgical tissues indicates a poor prognosis and a diminished likelihood of responding to platinum-based neoadjuvant chemotherapy [37, 38].

### Apoptosis

Most anticancer drugs aim to trigger cell death through various mechanisms, including apoptosis. If apoptosis is delayed or inhibited, resistance may develop, reducing the drug's effectiveness. Since chemotherapy is known to trigger apoptosis in cells, disruptions in the apoptosis pathway can result in resistance [39]. In drug-resistant ovarian cancer, the expression of anti-apoptotic proteins increases after treatment. These anti-apoptotic proteins can inhibit the initiation of apoptosis by directly or indirectly blocking the caspase cascade [9].

Mano et al. discovered that Bcl-2 expression correlated with inadequate response in ovarian cancer tissue samples from patients undergoing cisplatin-based treatment [40]. Yang et al. created chemoresistant SKOV3 and OVCAR3 ovarian cancer spheroids to elucidate the mechanism of platinum resistance in spheroids, demonstrated that spheroids exhibited higher Bcl-2 expression compared to ovarian cancer cells grown in monolayers [41]. This study illustrates the significance of Bcl-2 in ovarian cancer and its function as a gene that facilitates medication resistance by inhibiting apoptosis.

### Autophagy

Cisplatin therapy mechanistically stimulates ERK (Extracellular Signal-Regulated Kinase), hence facilitating autophagic cell death. Inhibition of ERK activation with MEK inhibitors or reduction of ERK expression through siRNA diminishes cisplatin-induced autophagy and therefore enhances the sensitivity of ovarian cancer cells to cisplatin-induced death [42]. In ovarian cancer cells that gain cisplatin resistance, both ERK activation and autophagy induction are elevated. Significantly, the suppression of ERK or the inhibition of autophagy enhances cisplatin-induced apoptosis in cells that have developed resistance to cisplatin. Consequently, ERK-mediated autophagy may induce resistance to cisplatin. The suppression of autophagy can surmount cisplatin resistance in ovarian cancer cells [42].

## The Relationship between Ovarian Cancer and Circadian Rhythm

### Circadian Rhythm

The circadian rhythm, or circadian clock, is an inherent time mechanism that regulates the biological processes of organisms in an approximately 24-h cycle. This rhythm governs the sleep–wake cycle, body temperature, hormone secretion, metabolism, and several physiological processes [43]. The circadian rhythm governs essential biological activities at the cellular level, including metabolism, DNA repair, the cell cycle, and apoptosis. The appropriate operation of these mechanisms is crucial for the healthy development and functioning of cells. Disruptions in circadian rhythm can result in anomalies that may contribute to cancer development [44]. The circadian cycle is aligned with environmental elements, including light. The suprachiasmatic nucleus (SCN) in the brain exerts central control. The SCN (Suprachiasmatic Nucleus) utilizes light signals perceived by the retina to regulate the circadian rhythm and disseminate these signals throughout the body. The circadian rhythm at the cellular level is regulated by the feedback loop of a set of genes referred to as 'clock genes' [43].

**Fig. 2:**
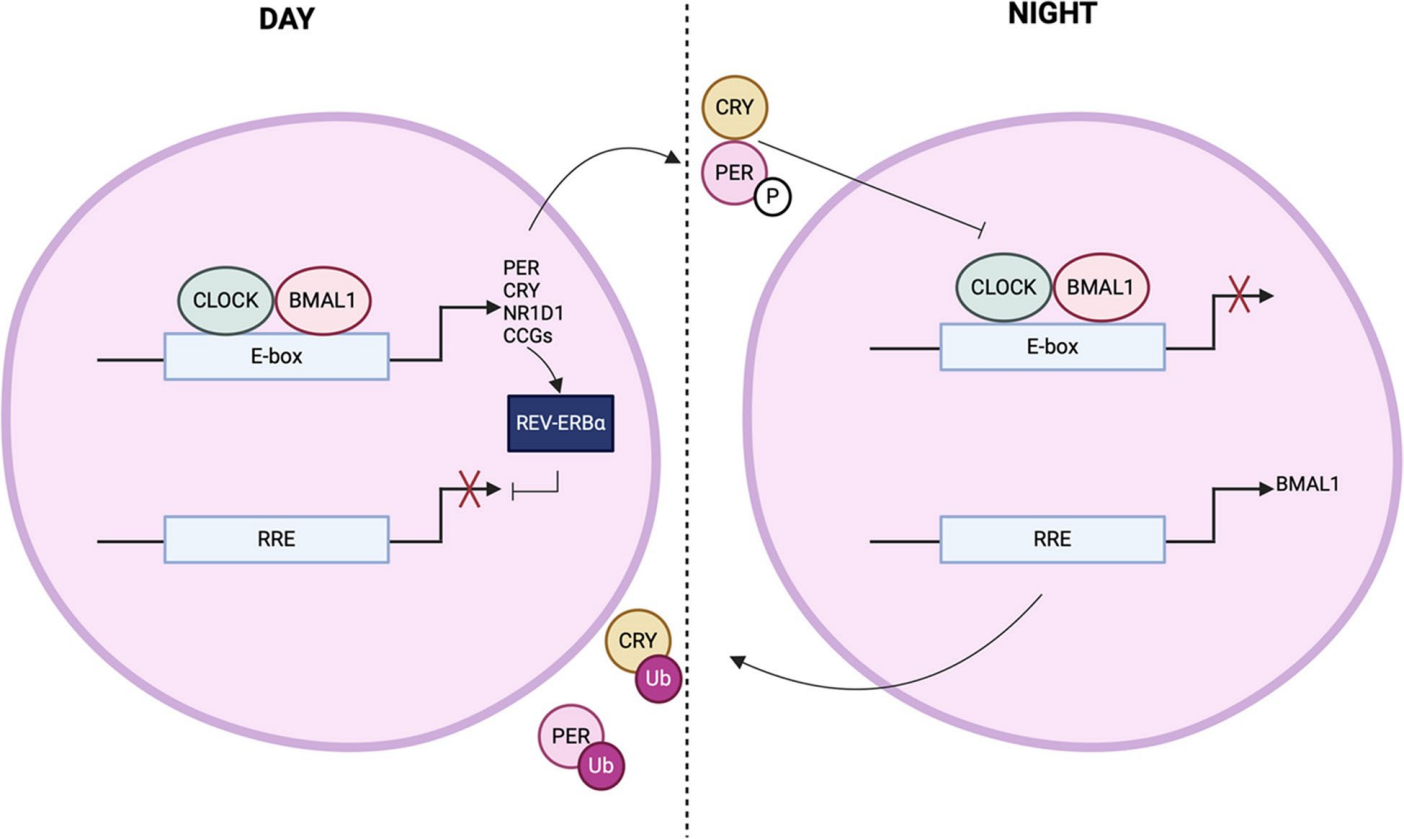
CLOCK and BMAL1 enhance the transcription of Period (Per), Cryptochrome (Cry), and other circadian rhythm genes (CCGs) during daylight hours. The concentrations of PER and CRY proteins grow at night, subsequently dimerizing and translocating to the nucleus to inhibit CLOCK-BMAL1-mediated transcription. PER and CRY proteins are then ubiquitinated and destroyed to commence a new circadian cycle. In contrast, REV-ERBα protein levels, encoded by NR1D1, are elevated throughout the daytime, during which it suppresses BMAL1 transcription. During the nocturnal period, REV-ERBα protein concentrations are diminished, facilitating BMAL1 transcription. P denotes phosphorylation; RRE refers to REV-ERB/ROR response elements; Ub signifies ubiquitylation

The peripheral cell autonomous circadian clocks, regulated by the master SCN, exist in every body cell and comprise a minimum of twelve genes. This encompasses the fundamental helix-loop-helix/PAS domain, which comprises transcription factors. The CLOCK (circadian locomotor output cycles kaput), BMAL1 (Brain and Muscle ARNT-Like 1), and NPAS2 genes initiate the transcription of circadian genes (PER1 and PER2- Period Circadian Regulators 1–2) and cryptochrome genes (CRY1 and CRY2) [45]. There are correlations between the cellular clock and the cell cycle. The molecular circadian clock influences cell cycle genes such as c-Myc, Wee1, cyclin D, and p21. Circadian rhythm is consequently associated with cancer [45]. The normal operation of these genes is crucial for the organism's health, and their disruption has been linked to numerous disorders [46] (Fig. 2).

### Effects of Circadian Rhythm on Ovarian Cancer

Research indicates that night shift employment correlates with a heightened incidence of both invasive and borderline ovarian tumors, especially the serous and mucinous subtypes, in women aged 50 and above [47]. Circadian genes are prominently expressed in the ovaries, where they govern ovulation. Circadian disturbance correlates with multiple risk factors for ovarian cancer. Jim et al. investigated single nucleotide polymorphisms (SNPs) in the circadian genes BMAL1, CRY2, CSNK1E, NPAS2, PER3, REV1, and TIMELESS, together with the downstream transcription factors KLF10 and SENP3, as predictors of epithelial ovarian cancer (EOC) risk and histological subtypes [48]. Their investigation revealed that the most notable relationship was rs117104877 in BMAL1. Functional study indicated a notable down-regulation of BMAL1 expression subsequent to cMYC overexpression and enhanced transformation in ovarian surface epithelial cells, alongside alternate splicing of BMAL1 exons in ovarian and granulosa cells. The findings indicate that variations in circadian genes, particularly BMAL1, may correlate with ovarian cancer risk, maybe via the disturbance of hormone pathways [48].

Recent multi-omics analyses have provided direct evidence that circadian clock genes themselves undergo epigenetic alterations in ovarian cancer. Zhu et al. [34] reported that several core clock genes, including ARNTL (BMAL1), CLOCK, CRY1/2, PER1/2/3, NR1D1, and RORA, display aberrant methylation patterns in ovarian tumors, which correlate with their reduced expression compared to normal ovarian tissue. Importantly, these epigenetic changes are not only associated with dysregulation of apoptosis, cell cycle, and DNA damage response pathways, but also with activation of oncogenic signaling such as RAS/MAPK and RTK pathways. Clinically, hypermethylation-driven downregulation of PER2 predicts poorer platinum sensitivity, whereas upregulation of PER3 and CRY1 expression correlates with adverse overall and progression-free survival. Moreover, mutations and epigenetic silencing of CLOCK and BMAL1 have been linked to impaired immune infiltration, further influencing therapeutic response. Collectively, these findings highlight that epigenetic regulation of circadian genes not only contributes to tumor progression but also impacts prognosis and chemoresistance, positioning them as potential biomarkers and therapeutic targets in ovarian cancer [34].

To systematically elucidate circadian clocks in ovarian cancer (OC), Zhao et al. [49] initially compared the mRNA expression levels of 12 established circadian genes—ARNTL, CLOCK, PER1, PER2, PER3, CRY1, CRY2, NR1D1, NR1D2, RORA, RORB, and RORC—in OC tissues and adjacent ovarian tissues utilizing the GEPIA (Gene Expression Profiling Interactive Analysis) online database. The expression levels of ARNTL, CRY2, NR1D1, PER1, PER3, and RORA were diminished, and the mRNA level of RORC was elevated in ovarian cancer tissues relative to normal tissues. This work demonstrates that fundamental circadian clock genes are extensively modified at the mRNA level in ovarian cancer [49].

## Resistance to Chemotherapy and Circadian Rhythm in Ovarian Cancer

Xu et al. assessed the expression of the CLOCK gene and protein in cisplatin-sensitive A2780 and cisplatin-resistant CP70 ovarian cancer cells subjected to varying doses of cisplatin [50]. They suppressed CLOCK expression in cisplatin-resistant CP70 cells using RNA interference. Following treatment with cisplatin, the growth of cisplatin-resistant CP70 cells was observed. CLOCK mRNA expression was considerably elevated in cisplatin-resistant CP70 cells relative to cisplatin-sensitive A2780 cells [51]. CLOCK protein expression was considerably elevated in cisplatin-resistant cells relative to susceptible cells. In conclusion, cisplatin treatment in both cisplatin-resistant and susceptible ovarian cancer cells demonstrated a dose-dependent augmentation of CLOCK protein expression correlating with elevated cisplatin concentration. After the suppression of CLOCK via RNA interference in cisplatin-resistant CP70 ovarian cancer cells, cisplatin treatment dramatically inhibited cell growth and induced apoptosis [51]. The expression of the circadian gene CLOCK in ovarian cancer cells was significantly correlated with cisplatin resistance. Downregulation of the circadian gene CLOCK in ovarian cancer cells may enhance sensitivity to cisplatin therapy.

Sun et al. [52] demonstrated that in SKOV3/CDDP (cisplatin-resistant) cells transfected with the RNA interference plasmid pSUPER-Clock and the CLOCK overexpression plasmid, the levels of autophagy and the number of cells in the G0/G1 phase were considerably reduced, while the expression levels of CLOCK, LC3, P-gp, and MRP2 were suppressed. Conversely, they demonstrated that the levels of autophagy and the number of cells in the G0/G1 phase were dramatically elevated, along with higher expression levels of CLOCK, LC3, P-gp, and MRP2 in cell lines transfected with pcDNA3.1-Clock [50]. Their findings indicated that, relative to the untransfected control group, the proportion of apoptotic cells in SKOV3/CDDP cell lines within the Clock-interfered expression group post-cisplatin treatment was markedly elevated, whilst survival rates were dramatically diminished [50]. The findings indicated that the suppression of circadian CLOCK gene expression influences drug resistance protein expression and may ultimately mitigate cisplatin resistance in the ovarian cancer SKOV3/CDDP cell line.

Wang et al. examined the function of PER2 in the responsiveness of ovarian cancer to cisplatin and the associated processes both in vitro and in vivo [52]. Protein levels of PI3K (Phosphoinositide 3-Kinase), AKT (Protein Kinase B), caspase-3, E-cadherin, and other drug resistance-associated markers were analyzed in SKOV3 and SKOV3/CDDP resistant cells, as well as in xenograft tumor tissues. The study revealed that in SKOV3/CDDP-resistant cells, PER2 expression was markedly diminished due to hypermethylation of the PER2 promoter. Conversely, PER2 overexpression enhanced cisplatin-induced death in both SKOV3 and SKOV3/CDDP cells while substantially suppressing their proliferation. Mice subjected to ovarian cancer xenografts derived from SKOV3/CDPP cells overexpressing PER2 exhibited a marked decrease in tumor mass relative to the control group. The overexpression of PER2 markedly reduced the protein expressions of PI3K, AKT, and MDR1, while enhancing the expressions of caspase-3 and E-cadherin in tumor tissues; conversely, the knockdown of PER2 exhibited opposing effects [52]. The findings indicate that diminished expression of the circadian gene PER2 in ovarian cancer cells is significantly correlated with cisplatin resistance.

**Fig. 3:**
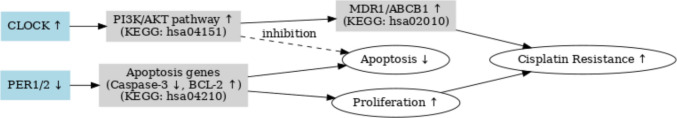
Mechanistic links between circadian clock genes, signaling pathways, and cisplatin resistance in ovarian cancer. Overexpression of *CLOCK* activates the PI3K/AKT signaling cascade (KEGG: hsa04151), which in turn upregulates the efflux transporter *MDR1/ABCB1* (KEGG: hsa02010), leading to enhanced cisplatin resistance and suppression of apoptosis. In contrast, downregulation of *PER1/2* reduces pro-apoptotic signaling (Caspase-3 ↓) while increasing anti-apoptotic factors (BCL-2 ↑) within the apoptosis pathway (KEGG: hsa04210). Loss of *PER1/2* also decreases E-cadherin expression, collectively promoting proliferation, survival, and drug resistance. The diagram highlights how circadian disruption influences both apoptosis and multidrug resistance pathways, thereby shaping chemotherapy outcomes in ovarian cancer

Wang et al. conducted a case–control study to examine the disparities in protein expression in tumor samples from ovarian cancer patients who engaged in night shift employment compared to those who did not [53]. They developed a model of circadian rhythm disruption associated with ovarian cancer in nude mice and examined the molecular mechanisms of circadian rhythm in the therapy of ovarian cancer. The night shift group had elevated expression of interleukin (IL)-6, programmed cell death receptor-1 (PD-1), and programmed death ligand 1 (PD-L1), while expression of tumour necrosis factor (TNF)-α, PER1, and PER2 was decreased. In conclusion, circadian clock disturbance adversely impacts chemotherapy efficacy in ovarian cancer and influences the expression of immunological factors and molecules associated with the phosphoinositide 3-kinase/protein kinase-B (PI3K/Akt) signaling pathway [53]. Mechanistic evidence links circadian dysregulation to PI3K/AKT signaling, MDR1 expression, and apoptosis in ovarian cancer. In mice, intestinal *mdr1a/Abcb1a* expression is directly clock-controlled via PAR-bZIP factors, providing a paradigm for circadian regulation of drug efflux transporters [54]. In ovarian models, *CLOCK* is elevated in cisplatin-resistant cells, and *CLOCK* knockdown restores cisplatin-induced growth inhibition and apoptosis [55]. Conversely, *PER2* shows tumor-suppressive activity: overexpression represses PI3K/AKT signaling, enhances apoptosis, and re-sensitizes SKOV3/SKOV3-DDP to cisplatin [52]; *mPer2* overexpression also induces apoptosis in other cancer contexts [56]. Beyond the core, *BMAL1* restrains invasion via the PI3K–AKT–MMP-2 axis [57]. Clinically, multiple clock genes (including *PER1*, *PER2*, and *CLOCK*) are downregulated in epithelial ovarian cancers versus normal ovary [58], and PI3K/AKT/mTOR signaling is widely implicated in ovarian chemoresistance [59]. These data justify depicting directional links from *CLOCK/BMAL1/PER2* to PI3K–AKT and apoptosis modules, and to efflux transporters (e.g., *ABCB1/MDR1*) in Fig. 3.

**Fig. 4:**
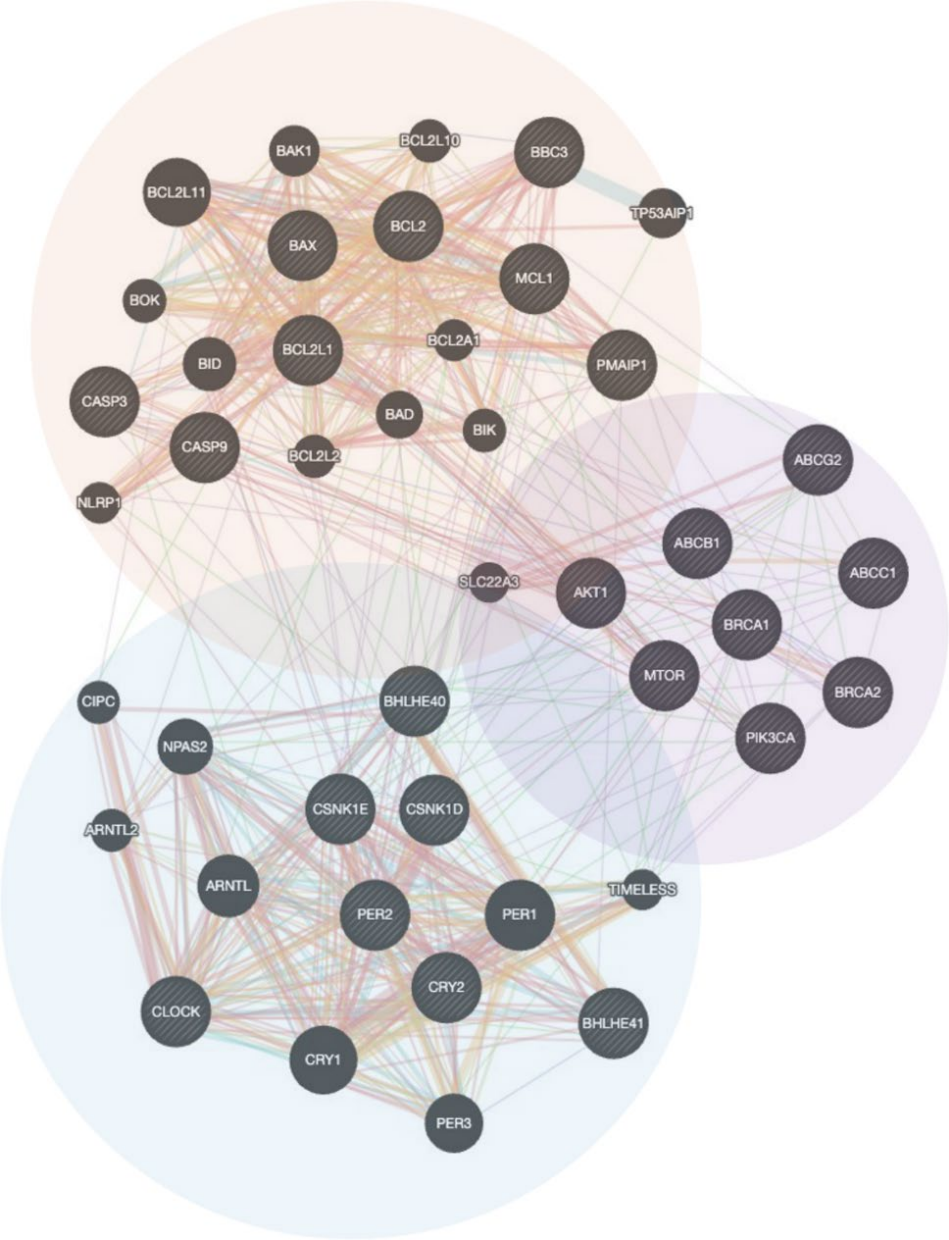
Circadian–apoptosis–chemoresistance network in ovarian cancer. Genes were grouped into three functional modules: circadian clock (blue; e.g., *CLOCK, BMAL1, PER2*), apoptosis regulators (orange; e.g., *BAX, CASP3/9, BCL-2, MCL1*), and chemoresistance/survival pathways (purple; e.g., *ABCB1, BRCA1/2, PI3K–AKT–mTOR*). Node fill colors indicate module assignment, and edge colors represent GeneMANIA evidence types: physical interactions (red), co-expression (pink), pathway (light blue), predicted (orange), shared protein domains (light green), genetic interactions (green), and co-localization (dark blue). Edge colors correspond to GeneMANIA evidence types, with relative contribution as follows: physical interactions (red, 53.18%), co-expression (pink, 15.69%), predicted (orange, 13.20%), pathway (light blue, 7.83%), shared protein domains (light green, 5.85%), genetic interactions (green, 3.09%), and co-localization (dark blue, 1.17%). The network illustrates how circadian disruption can impact apoptotic signaling and drug resistance mechanisms in ovarian cancer

Growing evidence indicates that circadian clock components are closely linked to apoptotic regulation and drug resistance in ovarian cancer. PER2 downregulation has been observed in ovarian tumors, and restoration of PER2 suppresses PI3K/AKT signaling, enhances apoptosis, and increases cisplatin sensitivity [52, 60]. Overexpression of PER2 has also been shown to induce apoptosis in cancer cells, supporting its tumor-suppressive role [56]. Similarly, BMAL1 suppresses invasion by blocking the PI3K/AKT/MMP-2 pathway [57]. In ovarian cancer specifically, CLOCK upregulation has been associated with proliferation, invasion, and cisplatin resistance, while its knockdown restores apoptosis and drug sensitivity [55]. Clinical data further suggest that reduced expression of clock genes, including PER1, PER2, and CLOCK, correlates with advanced stage, poor prognosis, and decreased chemotherapy response [58]. Moreover, the circadian system directly regulates drug transporters, as intestinal mdr1a (ABCB1 homolog) expression exhibits circadian rhythmicity, linking molecular timekeeping with efflux-mediated drug resistance [54]). Collectively, these findings outline a mechanistic framework in which circadian disruption alters apoptotic balance and activates chemoresistance pathways, ultimately shaping therapeutic response in ovarian cancer (Fig. 4).

## Conclusion and Suggestion

Ovarian cancer, a predominant cause of cancer-related mortality, presents considerable challenges in detection and treatment owing to its insidious and silent development, coupled with nonspecific symptoms. Treatment encompasses surgical intervention, chemotherapy, and targeted therapies. Chemoresistance in ovarian cancer is a multifaceted challenge that significantly impairs treatment outcomes. Various processes, including the disturbance of the circadian clock, contribute to the development of this resistance, and a comprehensive understanding of these pathways is crucial for formulating successful therapeutic options. Disruption in circadian rhythm has been linked to the onset of various health issues, including ovarian cancer. Nevertheless, limited research has been undertaken about circadian rhythm modification resulting from treatment resistance in ovarian cancer. Enhancing understanding of the molecular pathways linked to the disrupted circadian clock and its components in ovarian cancer may facilitate the development of more effective medications and treatment strategies for the illness.

## Data Availability

The data that support the findings of this study are available on request from the corresponding author.

## Abbreviations

ABC: ATP-binding cassette
ABCB1: ATP-binding cassette subfamily B Member 1 (P-glycoprotein, MDR1)
ABCC1/2: ATP-binding cassette subfamily C Member 1/2 (MRP1/MRP2)
ABCG2: ATP-binding cassette subfamily G Member 2 (BCRP)
AKT: Protein kinase B
AR: Androgen receptor
ATP: Adenosine triphosphate
ATP7A/B: ATPase copper-transporting alpha/beta
BAX: BCL2 associated X, pro-apoptotic gene
Bcl-2: B-cell lymphoma 2
BCRP: Breast cancer resistance protein
BMAL1 (ARNTL): Brain and muscle ARNT-like 1
BRCA1/2: Breast cancer gene 1/2
CCG: Circadian clock genes
CDDP: Cisplatin (cis-[PtCl₂(NH₃)₂], DDP)
c-Myc: Cellular myelocytomatosis oncogene
CRY1/2: Cryptochrome circadian regulators 1/2
CSCs: Cancer stem cells
CSNK1E: Casein kinase 1 epsilon
DDP: Cisplatin (alternative abbreviation)
DNA: Deoxyribonucleic acid
E-cadherin: Epithelial cadherin
EOC: Epithelial ovarian cancer
ERK: Extracellular signal-regulated kinase
FDA: Food and Drug Administration
GEPIA: Gene expression profiling interactive analysis
GSH: Glutathione
GSTP1: Glutathione S-transferase P1
HDAC1/6: Histone deacetylase 1/6
HGSOC: High-grade serous ovarian cancer
HIPK2: Homeodomain-interacting protein kinase 2
HLF: Hepatic leukemia factor
HNPCC: Hereditary nonpolyposis colorectal cancer
hnRNPA2B1: Heterogeneous nuclear ribonucleoprotein A2/B1
HRR: Homologous recombination repair
IL-6: Interleukin-6
KLF10: Krüppel-like factor 10
LC3: Microtubule-associated proteins 1A/1B light chain 3B
LRP: Lung resistance protein
MAPK: Mitogen-activated protein kinase
MDR1: Multidrug resistance protein 1
miRNA / miR: MicroRNA
MMR: Mismatch repair
MRP1-7: Multidrug resistance-associated proteins
MT: Metallothionein
mTOR: Mechanistic target of rapamycin
NACT: Neoadjuvant chemotherapy
NER: Nucleotide excision repair
NPAS2: Neuronal PAS domain protein 2
OC: Ovarian cancer
p21: Cyclin-dependent kinase inhibitor 1A (CDKN1A)
PARP: Poly (ADP-ribose) polymerase
PD-1: Programmed cell death receptor-1
PD-L1: Programmed death ligand-1
PER1/2/3: Period circadian regulators 1/2/3
PgP: P-glycoprotein
PI3K: Phosphoinositide 3-kinase
RORA/B/C: RAR-related orphan receptor A/B/C
RRE: REV-ERB/ROR response elements
SCN: Suprachiasmatic nucleus
SENP3: SUMO-specific protease 3
SNP: Single nucleotide polymorphism
STIC: Serous tubal intraepithelial carcinoma
TAMs: Tumor-associated macrophages
TGF-β: Transforming growth factor beta
TME: Tumor microenvironment
TNF-α: Tumor necrosis factor alpha
Ub: Ubiquitylation
UTR: Untranslated region
YAP1: Yes-associated protein 1
